# Explicating the work of regional leads in implementing at scale: a qualitative study of the perinatal optimisation care pathway

**DOI:** 10.1186/s43058-026-01061-3

**Published:** 2026-08-06

**Authors:** Julie Roberts, Josie Anderson, Natalie Armstrong, Elaine Boyle, Penelope McParland, Thomas Padden, Carolyn Tarrant, Janet Willars, Nicola Mackintosh

**Affiliations:** 1 Division of Public Health and Epidemiology, School of Medical Sciences, University of Leicester, University Road, LE1 7RH Leicester, Great Britain; 2 Bliss, Union Street, SE1 1SZ London, UK; 3 School of Health & Medical Sciences, City St. George’s, University of London, EC1V 0HB London, UK; 4 University Hospitals of Leicester NHS Trust, Leicester, UK; 5 School of Healthcare, University of Leicester, University Road, LE1 7RH Leicester, UK; 6 National Institute for Health and Care Research (NIHR) Greater Manchester Patient Safety Research Collaboration (GM PSRC), The University of Manchester, Manchester, M13 9PL UK

**Keywords:** Quality improvement, Optimisation, Normalisation process theory, Health innovation networks, Maternity care, Neonatal care, Qualitative research, Communities of practice, Cognitive participation

## Abstract

**Background:**

Spread and scale-up of evidence-based interventions in healthcare is complex, given the challenges of different contexts, networks of interactions and collaborative processes in healthcare delivery. Quality Improvement Collaboratives (QICs) provide a multi-organisational structured approach to improvement. However, the current evidence base underpinning QICs effectiveness is still limited and effectiveness appears strongly bound to context. Seeing scale-up and spread as a social practice, this paper explores the work of Patient Safety Collaboratives (PSC) in England that support implementation of a nationally mandated perinatal optimisation care bundle.

**Methods:**

The PremPath study explored implementation of perinatal optimisation at scale across different regions within England. We report from the first work package, comprising scoping interviews with 40 strategic leads from across Patient Safety Collaboratives (PSCs) in England who support implementation and quality improvement of perinatal optimisation. Analysis drew on the constant comparison method and Normalisation Process Theory (NPT) to explore implementation activities and contextual challenges.

**Results:**

Findings demonstrate cognitive participation as a key feature of the improvement work undertaken at regional level. This entailed bringing healthcare staff together into a multi-disciplinary community of practice focused on the pathway. Data work underpinned efforts to identify priorities for improvement and track progress. However, fluctuations in performance contrasted with the ideal of continuous improvement. Regional leaders identified turbulence in the context of maternity and neonatal care as contributory factors to fluctuations. Our work highlights, for regional leads, the hidden emotional work and micropolitics of engaging clinical staff in communities of practice and shifts attention from individual implementation efforts to the broader context of maternity and neonatal care, which presented as dynamic, unstable and already crowded with other improvement priorities.

**Conclusions:**

Our findings provide an overview of the landscape of perinatal optimisation at regional level in England, helping to open up the ‘black box’ of collaborative efforts managed via national PSCs. Our work highlights the significance of the micropolitical dimensions of regional leads’ improvement work, and raises questions around how best to ensure sustainability of change given the current inelasticity of the implementation environment of maternity and neonatal care.

**Supplementary Information:**

The online version contains supplementary material available at https://doi.org/10.1186/s43058-026-01061-3.

Contributions to the literature

- This study contributes to recognised gaps in the literature regarding the work of regional leads within improvement collaboratives, when undertaking implementation at scale.
- Within the context of perinatal optimisation, we show how the improvement work undertaken by regional leads, via their local communities of practice, was shaped by dynamic elements of the maternity and neonatal context and the specifics of the optimisation pathway being implemented.
- We draw attention to micropolitical dimensions of regional leads’ improvement work, notably managing improvement in the context of workforce shortages and competing priorities, which is often missing in accounts of implementation at local level.

## Background

Spread and scale-up of evidence-based interventions in healthcare is complex, given the challenges of different contexts, networks of interactions and collaborative processes in healthcare delivery [1]. Rather than taking an approach based on a mechanical logic that emphasises standardisation and replicability, it requires attention to the underlying ecological logic of complex systems [2, 3]. Seeing scale-up and spread as a social practice enables us to attend to what is understood as ‘salient, meaningful, ethical, legal, materially possible, and professionally or culturally appropriate in particular social situations’ [4].

Spread and scale up, adapted to local context, are an important component of quality improvement (QI). QI ‘refers to practical efforts to make changes to healthcare configuration and delivery, usually in the form of discrete QI projects utilising QI tools and methodologies’ [5, 6]. Quality Improvement Collaboratives (QICs) are increasingly used in high-income countries to accelerate improved outcomes [7]. They vary in configuration but share a multi-faceted, multi-disciplinary approach to improvement with five essential features: a specified topic; membership with clinical and quality improvement (QI) expertise; involvement of multi-professional teams across different settings; use of an improvement framework (setting targets, collecting data and testing changes); and use of a structured collaborative process [7]. By enhancing learning environments in this way, QICs address local implementation problems, and increase the spread and implementation of ‘best practices’ across settings [8, 9]. However, the current evidence base underpinning QIC’s effectiveness in spreading best practice is limited [9–12] and effectiveness appears strongly bound to context [10], including project leadership and access to data, the wider organisational context and external environment (e.g., external incentives, QI culture) [13].

One example of a QIC is the Patient Safety Collaborative (PSC), established in England in 2014 to spread best practice and support continual learning, safer cultures and safe systems within the National Health Service (NHS) [14]. There are 15 regionally-based PSCs, funded and nationally coordinated by NHS England, which drive cultural and system change, support evidence-based QI methodology and build local QI capability. PSCs deliver on their remit through the Health Innovation Networks (HINs), whose role is to support implementation of interventions at scale and the spread of innovation in healthcare. HINs are conceptualised as a network of networks [15]. Decentralised management, vertical accountability and negotiation are key features of HINs [15], in line with the concept of continuous improvement, which seeks to reject blame culture and shift focus to understanding why systems produce particular outcomes and how they can be improved [16].

The work of PSCs is shaped by both national and local priorities. Nationally-mandated priorities come from the National Patient Safety Improvement Programmes. These are an important element of the NHS Patient Safety Strategy in building a safe healthcare system [17], and included, at the time of our research, a focus on maternal and neonatal safety. The Maternal and Neonatal Safety Improvement Programme (MatNeoSip)– aims to improve outcomes and reduce unwarranted variation across maternity and neonatal settings. Perinatal optimisation is currently one of two core workstreams in the programme. Led by regional workstream leads employed by the PSCs, MatNeoSip aims to spread and improve adoption of a perinatal optimisation care pathway, set out in *Saving Babies Lives v3* [18].

**Fig. 1:**
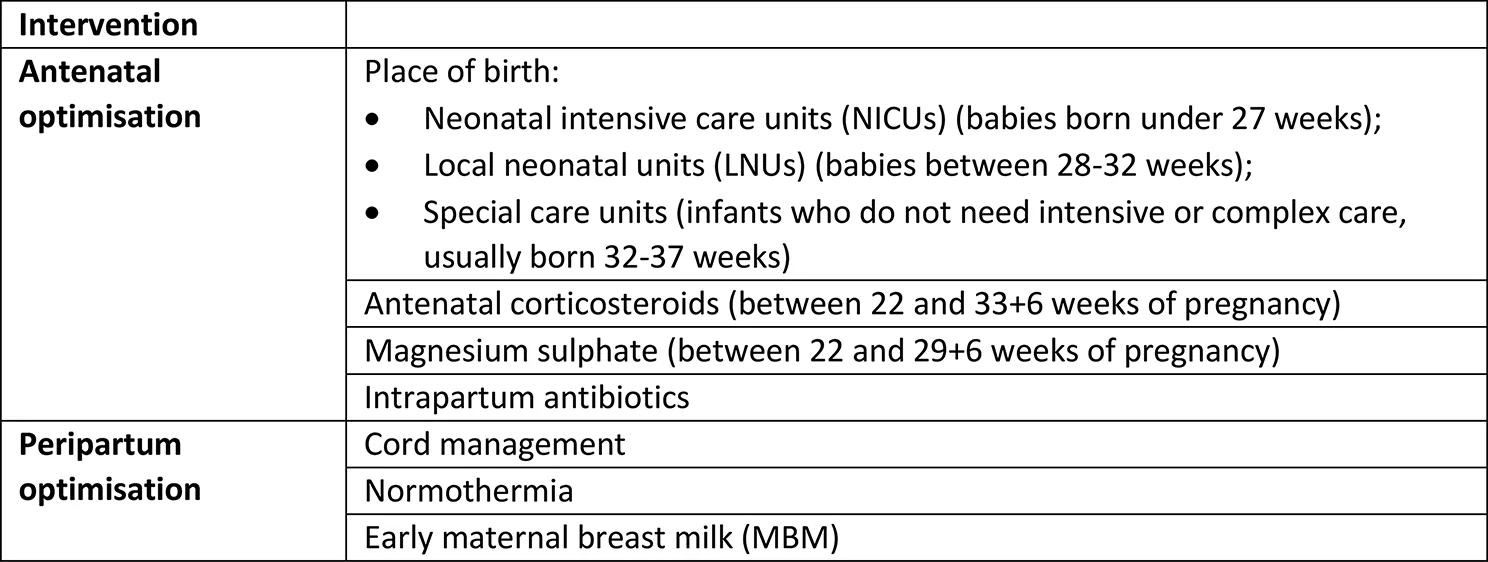
Evidence based interventions for optimisation and stabilisation of the preterm infant. These 7 interventions were the focus of our research: additional interventions have since been added

The care pathway for the optimisation and stabilisation of the preterm infant was set out by the British Association of Perinatal Medicine (BAPM) to reduce the risk of neonatal death and associated preterm morbidities through inclusion of specific evidence-based interventions. The pathway spans maternity and neonatal care, requiring increased emphasis on collaborative working across services. Different regions adopted different versions of the pathway, included branded pathways such as PeriPrem and Prem7, although all include the core elements of the BAPM pathway. Figure 1 shows the core elements of the pathway.

**Fig. 2:**
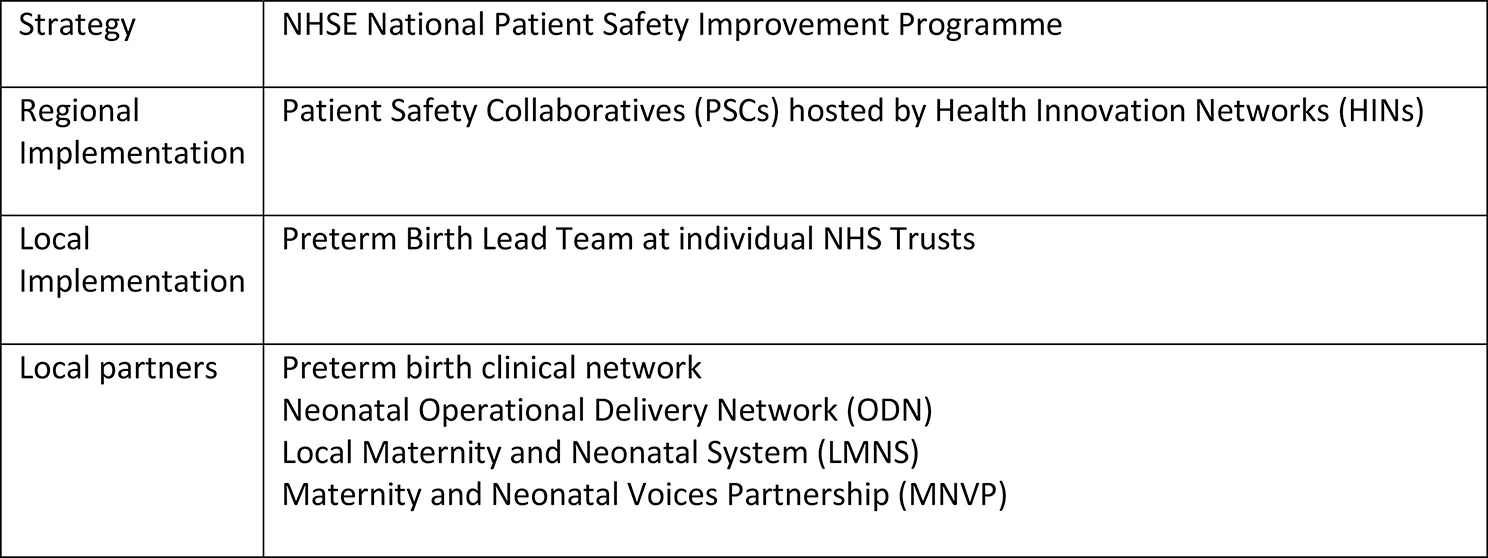
Organisations responsible for implementation of *Saving Babies Lives v3*

The pathway is set out in *Saving Babies Lives v3* (SBL3). All maternity service providers are responsible for implementing *Saving Babies Lives v3.* Local Maternity and Neonatal Systems and Neonatal Operational Delivery Networks work together to coordinate maternity care and specialized neonatal medical services. They work with Patient Safety Collaboratives to deliver on the NHSE commissioned safety improvement work. Figure 2 shows national, regional and local organisations responsible for implementation. SBL3 *takes* a ‘continuous improvement’ approach which requires routine measurement, increasingly taken to be necessary to achieve improvement in healthcare [16]. Neonatal units collate and report statistics about how many eligible mothers and babies receive each element of the pathway.

A need for further evaluation of large-scale improvement initiatives in maternity and neonatal care in the NHS has been identified [19]. Evaluation of a previous iteration of Saving Babies Lives (v2) has identified improvement in a number of process measures and reduction in stillbirth rates over the evaluation period [20]. Other research has evaluated QI programmes targeting implementation of magnesium sulphate as neuroprotection for preterm infants, now one element of the full pathway. This has produced evidence of effectiveness of magnesium sulphate [21–23] and generated important insights about scale and spread [24, 25]. The general principles of the PSC approach at regional level is known, i.e., periodically convening members of the network, establishing shared goals, and providing infrastructural support to their local Trusts [9]. However, there is a paucity of data exploring the situated work of regional leads and the specifics of the maternity and neonatal context.

This paper addresses the questions: What work do PSC regional leads undertake to implement the optimisation pathway at scale? How do they manage complexity arising both from the pathway itself (e.g. its multiple components) and the specific context of maternity and neonatal care? We focus at regional level, rather than assessing ability of Trusts’ locally to enable perinatal optimisation, to deepen understandings of how regional leads work with these complexities to drive improvement, in ways that show sensitivity to the realities of the maternity and neonatal environment and the ‘optimisation’ task at hand. This paper answers calls for further research into processes and practices of improvement within PSCs [14, 26] and draws attention to key features of the social and micropolitical dimensions of improvement work at regional level [27], which is often missing in instrumental accounts of implementation [28, 29].

## Methods

The work reported here presents the first phase of a larger, ethnographic study examining how the perinatal optimisation care pathway for preterm infants was being implemented in England. The study was informed by an interpretivist research paradigm. We draw on Normalisation Process Theory (NPT), understanding implementation as a co-constructed and iterative process which shapes, and is shaped by, the social organisation of work [30, 31].

The study was undertaken by a multi-disciplinary team including social scientists, an obstetrician, a neonatologist, and a parent, working alongside a panel of six parents of preterm infants and an expert advisory group of healthcare practitioners, policymakers and academics. This ensured that multiple perspectives enhanced the quality of the research and the relevance of the findings to healthcare policy and practice.

### Sampling

A purposive sample of maternity and neonatal workstream leads within all 15 regional PSCs was identified by NHS England who circulated a call for participants via email, and a covering letter assuring the recipient of the independence of the research and their right to decline. Potential participants were invited to contact the research team directly to express interest. On contact, potential participants were provided with a participant information sheet and consent form and invited to ask any questions. Once workstream leads were recruited, snowball sampling was used to recruit other key people within regional configurations, including patient advocates, clinical staff with roles in implementing the pathway or improving local practice, and clinical governance staff. Interviews took place between July 2023 and February 2024. Our sample size of 40 was guided by information power and aimed to generate sufficient quality data to produce new insights that could contribute substantially to current understanding [32].

### Interviews

Written consent was received from participants via email in advance and verbally confirmed at the start of each interview. A topic guide was informed by the study aims and literature review, and agreed by the research team, including parent representation. Interviews focused on local configurations of the care pathway and the context of service provision. Semi-structured interviews were conducted remotely and recorded within MS Teams. Interviews were conducted by Roberts and Willars, experienced, postdoctoral, qualitative health researchers. Each interview lasted up to 60 min.

Interviews were transcribed by a professional transcriber, anonymised at transcription, and allocated a unique identifier. Identifiers included the PSC region in order to be able to maintain links between multiple points of view within regions. Transcripts were uploaded to NVivo 11 for data management and coding.

### Analysis

Analysis was conducted by Roberts with support from the wider team. Analysis was informed by the constant comparison method [33]. Using a blended approach, coding was both deductive and inductive. Deductive coding was based on the research questions, the components of the care pathway and normalisation process theory (NPT) [30, 31].

NPT is a model, with roots in social science, of how complex practices become workable and embedded in context. The focus is on both individual and collective behaviour. NPT distinguishes between implementation contexts, mechanisms and outcomes. In this paper, we focus on implementation mechanisms as this describes the collaborative work required to achieve the goals of an improvement initiative. It has salience for complex interventions in healthcare settings where it provides a conceptual frame to describe and explain mechanisms that shape implementation processes [34]. Four further constructs are identified as part of implementation mechanisms: coherence building; cognitive participation, collective action and reflexive monitoring. We conceptualise the approach of the PSCs in NPT terms as ‘cognitive participation’. Cognitive participation refers to ‘how people work together to create networks of participation and communities of practice around interventions and their components’ ([31], p5).

Inductive codes were allocated to salient data points that were not accounted for by deductive codes derived from NPT, and clustered around the context of implementation and relational work of regional leaders.

Inductive and deductive coding are reported together here.

## Results

Forty participants were recruited from 12 of the 15 PSC/Health Innovation Networks. Three PSCs did not respond to invitations to participate. Twelve workstream leads took part; twenty-eight further participants were in leadership roles in the regions. Participants came from a range of professional backgrounds: midwifery, nursing, obstetrics, neonatology and non-clinical background such as parent advocates. Table 1 shows interview participants by professional background.

**Table 1:** Participants by professional background. Totals do not match number of participants as some participants had more than one professional background

Professional background	Workstream leads	Other regional leaders	Totals
Nursing	5	7	12
Midwifery	6	5	11
Obstetrics	1	4	5
Neonatology	1	4	5
Non-clinical	1	4	5
Other		2	2

Our data illustrate the significance of cognitive participation as a key mechanism underpinning regional leads’ efforts to enable the integration and embedding of pathway interventions into local everyday practice. Cognitive participation for the leads involved initiation, enrolment into the community, legitimation of the improvement work, and activation to maintain momentum. Following this framework, the first theme describes the activities that regional leads undertook to build, support and sustain communities of practice dedicated to perinatal optimisation. Importantly, we then describe how regional leads’ work to support implementation was shaped and situated, in the context of this specific programme and healthcare setting. We consider ‘the problem of context not as a fixed organisational structure or institutional entity but as an unstable, unfolding, process’ ([35], p5). We present two additional themes: intervention plasticity and contextual inelasticity; and relational and emotional work.

### Building and sustaining communities of practice for cognitive participation

Regional leads described their role in supporting pathway implementation across their regions. Reflecting the boundary spanning required to deliver the pathway effectively, much of their work involved creating forums to bring people together regularly as a means of building a community of practice. Forums typically included people from across the region, different professional groups, and sometimes representatives from service-user groups. Workstream leads recognised the need to cater for multi-disciplinary groups.

it’s no good having a programme where there’s no neonatal element because they [neonatal colleagues] simply won’t turn up’ (002 Workstream lead).

Key features of their work to build communities of practice, and support these communities in pathway implementation included: providing QI expertise; harnessing audit data to prompt action; creating safe spaces to enable shared problem solving and manage knowledge contestations; and legitimising the pathway through evidence and bringing in parent voices; sharing good ideas and effective implementation strategies, and promoting equitable contributions.

Workstream leads reported having training in QI methodology. They used those skills to support improvement efforts in their region. This could include adapting implementation tools for regions, or providing additional QI expertise for local teams:

We had the national driver diagrams within the toolkits, and then we developed a regional driver diagram, and then Trusts developed their own if they wished to do so. (008 Workstream Lead)

sometimes we’re […]sort of challenging people […] being that critical friend in quality improvement […] each trust has always got quality improvement people. So what we do is find out […] where are they on their level of understanding about QI, where do we need to support them to improve that level. (005 Workstream Lead)

Data work [36] underpins quality improvement at regional and local level. As one workstream lead described, ‘data underpins everything we do!’ (003 Workstream lead). Regional leaders described the importance of routine work that they undertook within local regions, to collate and review audit data from across the region’s units. This enabled identification of areas for improvement across the region or within individual Trusts and enabled the community to monitor change over time.

Consensus around the value of the pathway appeared to be high but some legitimation work was needed to reinforce the value of individual elements of the pathway and address areas where evidence was contested. A safe space within the community of practice was important to enable staff to participate openly in discussions around implementation of the pathway and enable collective problem solving.

They’re very open, very honest [discussions], people feel safe to say we don’t do that […] because we didn’t know we were supposed to do it. […]And then you’ll get some people say we don’t do it because they don’t want to do it. But that open and honest debate has actually led to improvements […] (022 Public Member).

It’s a very relaxed safe space where we can […] pose a problem, somebody will […] come and say actually […] our time from a woman presenting as possibly in…preterm labour, to actually having that diagnosed, […] there’s sometimes a two-hour delay because actually there’s […] no medic available to undertake a speculum examination, and midwives aren’t signed off as competent typically for preterm speculum examinations […] and so that was a problem that we pulled apart and actually we were able to identify that up in [another location] they […] developed a competency framework for midwives to be signed off. So we were able to find that and share that then amongst our community, so they can bring that back to their organisations. (016 Workstream Lead)

Legitimation work also involved discussing the evidence, sharing narratives of improved outcomes between Trusts, collating evidence sources online, or inviting external speakers, sometimes including parents, to share their knowledge and expertise.

We were able to access a whole range of academic speakers to come and deliver those sessions. (025 Project Manager)

We do try and make sure that we incorporate that user voice into any educational events that we put on. (006 Workstream lead)

I said [national expert], please can you come talk to us about this, because here’s what our doctors and midwives and nurses are saying, they don’t want to aim for this, we think this is not right […] he delivered a webinar called use and misuse of steroids. It was perfect. It could not have been more perfect. (002 Workstream Lead)

Regional leaders noted the significance of networking and sharing resources within their communities of practice. One participant described her work as ‘I lift and shift ideas’ (010 Consultant Neonatologist). Our data showed leads used enrolment strategies by inviting teams that were successfully implementing one or more elements of the pathway to present their work at regional meetings, or by allocating ‘buddies’ to organisations that were struggling with an element of implementation, as described below:

We just shared round what we’d heard and how to overcome it, and also took the data of the best performing units and buddied them up with the ones that were the least performing, and said you guys need to have a chat, ‘cause they’ve worked out how to do this (019 Programme Manager).

Part of regional leaders’ enrolment strategies involved ensuring equity between clinical disciplines and between units in agreeing regional practices:

I think that helping people feel involved in the group as well, like making everybody take part in it, making sure everybody’s voice is heard. Everybody contributed to the development of the guideline and I think it was important to everyone that we try and standardise care as much as possible. (011 Consultant Obstetrician)

### Intervention plasticity and contextual inelasticity

We turn now to consider how the dynamic elements of the maternity and neonatal context intersected to shape the improvement work of regional leads.

Interventions are often designed with a number of components, but characteristically these have a degree of plasticity [35], often with a central component (the hard core) allowing for discretionary adaptation of the soft periphery [27, 37]. The perinatal optimisation pathway presented multiple layers of operational complexity, creating challenges for regional leads to ensure the pathway was workable and integrated in its operational context. They had to constantly work to keep momentum going for the full pathway. However, to make it manageable, regions typically identified between one and three elements of the pathway to focus on at a time via their communities of practice.

There’re times when […] I need to drill down to one […] element or innovation at that certain point, because you look at data and the data’s telling you that you might need to just have a more deeper look into what is happening around that (005 Workstream Lead).

Magnesium we weren’t doing so badly, but I think for some reason [the adherence rates have] crept down recently. And I’m not quite sure why that is [...] With this you have to always keep that kind of momentum up (033, Lead Nurse ODN).

In contrast to the notion of continuous improvement, regional leads found themselves constantly appraising reasons for variation in performance, some of which were more easily identifiable than others, and some with multiple contributing factors which were more or less modifiable.

In quarter two last year you was achieving […] 90% […] or you was achieving six out of the seven elements […] But then in quarter three you had real big troughs. Why is that? […] Is it a changeover in your junior staff? […] was the induction correct for them? Did they know about [perinatal optimisation]? […] we should be seeing a steady line of these interventions, but we’re not, we’re seeing peaks and troughs. (003 Workstream Lead)

We still get peaks and troughs […] For example, we had a Trust that had an unprecedented number of babies born before arrival, which affected the normothermia rates for them quite significantly. (019 Programme Manager)

Given this fluctuation, some regional leads chose to opt for a pragmatic approach to achieving optimisation, setting their sights on consistently achieving minimum standards for the measures.

The national team this year gave us a range of parameters to try and achieve as a minimum; their upper expectation or stretch target would have been X [...] Maybe the minimum is where we can try to sustain at, rather than see dropping off. (016 Workstream Lead)

Turning now to the properties of the maternity and neonatal environment within which the pathway was operating, regional leads described an ‘inelastic context of practice’ [35]. Whilst regional leads could maintain a focus on improvement efforts around optimisation, clinical staff were also juggling other priorities and demands on their time.

I’ve definitely been frustrated that change is quite slow sometimes, but I’ve got to remember that…Preterm is my whole life, but it’s not everybody’s whole life, because they’ve got so many other things being thrown at them all the time, that they’re being told [are] equally important. (012 Preterm Labour Midwife)

I cannot underestimate the work that is being done on the ground, and one of the issues is because [...] everything is a priority. So we’ve had the Ockendon Report, we’ve had East Kent Report, we’ve had three-year delivery plan. We’ve had the CNST, so every time there’s something, and it’s all priorities that need to be actioned, and then you’ve got as well me coming and saying we also need to make sure this is a priority [...] I do admire how they’re even managing to make sure that they keep on top of everything. (005 Workstream Lead)

Service pressures, shortages and high turnover of staff in maternity and neonatal settings impacted on regional leads’ negotiating capacity.

I think it’s just been gaps in rotas and having to cover lots of extra clinical services, and so whenever we have a meeting [the consultants] are always covering something else. And so it’s really difficult to get them to come, and I suppose unless they appoint three more consultants […] it’s not gonna get any easier. (011 Consultant Obstetrician)

You’re gonna probably hear the same thing from everyone on this one: chronic workforce shortages. The workforce situation is the worst it’s ever been, absolutely, in terms of engagement […] No-one is being released to do anything. Free training is being put on […] staff can’t be released, it’s not a priority. (019 MatNeoSip Programme Manager)

### Doing relational and emotional work to sustain communities of practice

Workstream leads described the affective dimensions of working *responsibly* and *ethically* to bring people into the community of practice and to maintain engagement, in the context of the precarity in which they were operating. Levels of engagement with PSCs and regional structures varied. Workstream leads in the HINs have no ‘position power’ [38, 39] to compel Trusts to engage with the support they provide and so regional leads employed a variety of soft strategies to exert influence. Sometimes this involved making themselves available and relying on clinical staff to recognise the value of the collaboratives and take the first step, with varying levels of success:

[Y] ou can’t impose yourself on Trusts because they’ve got a million priorities. You kind of wait for the invitation. [...] I’m constant without being too annoying. (002 Workstream Lead)

We put a general offer of support out there, saying if anybody needs any help with this, or to look at the data, please contact us. Now as a general rule people don’t and we’re not really quite sure why that is. (006 Workstream Lead)

To a certain extent, the work of senior leaders was dependant on their levels of influence and social capital. Their role was facilitated by them being well-networked, trusted and seen as knowledgeable. Their reputation might be achieved over a career of working in different parts of a region and in different roles, but also by conscious efforts to build and maintain relationships.

So I have an excellent…advanced neonatal nurse practitioner…she’s very experienced. She also works as part of the retrieval team, so she’s part of the team that go out to collect preterm babies…so she knows all of the network, she knows the neonatal teams, and that’s really important (002 Workstream Lead).

Building relationships happened not just within regional meetings, but with visits to Trusts, individual phone calls and informal meetings. Relationship building was easier in smaller regions where the key clinicians were evident, where staff turnover was lower and relationships could be maintained over time.

We are not a network of 15 or 30 trusts, We can almost put our… Your arms around our Network…So when you have that number you can do a lot, if you have that drive and the enthusiasm (002 Workstream lead).

it is more of a problem, turnover in our area than anywhere else, but… the leads leave and you have to find somebody else and get them up to speed (001 Workstream lead). However, regional leaders laboured to exert their influence sensitively. Part of their relational work involved understanding and working *dynamically* with the context within which the perinatal optimisation care bundle was being implemented.

I’ve been very concerned in our Trust that we take this steady, that we make sure that we’re not overburdening our teams. So that we keep the engagement. So any kind of push to move on before we’re ready I’ve firmly resisted. (002 Workstream Lead)

I’m sometimes a bit of a bull in a china shop, so I’m trying to be gentle. (012 Midwife)

‘Personal power’ [39] involves utilising charisma, energy and enthusiasm to enable engagement and implementation. Regional leads often exerted this themselves but also noted that good working relationships with local champions with personal power were central to spreading good practice. Champions were valued for their passion and ability to influence other staff.

[name] for example, she’s a consultant paediatrician, absolute superstar. I regularly tell her, and […] a really important safety lead, [name]. Those two people, everybody else will fall in. (002 Workstream Lead)

However, leaders recognised the risk of over-burdening these few key individuals. They described managing tensions between maintaining momentum with improvement efforts and attending to individuals’ wellbeing.

you’re often concerned that it’s the same people doing the same work and you don’t want them to get burdened or burnout because they’re the go-to person, but you’re then grateful that you’ve got a go-to person! (021 Workstream Lead)

Some PSCs’ allocated funds to backfilling roles and freeing time for staff to engage with regional improvement activities, but this funding tended to be time-limited leading to reduced engagement once it came to an end.

Trusts received just over £1,000 […] they had an understanding give us a clinician’s name, who would directly work with me. So it would be somebody that I could go to, to say, have you looked at your data this month? What do you think the issues are? Could you give me some intelligence behind that? […] I’ve been having weekly calls with the [optimisation] links, they have tailed off considerably, because our funding came to an end. (003 Workstream Lead)

Regional leaders also described ethical dimensions of accommodating staff’s difficulties sustaining improvement at Trust level. Interviewees emphasised that frontline staff wanted to deliver good care and were ‘doing their best’ in health service under pressure. Regional leads described working sensitively to avoid adding to the burden on staff. They tried to ensure they were not complicit in contributing to the ethical load experienced by staff.

it’s quite easy to be on the sidelines and think oh, well they didn’t manage to do that, but how can we judge when we’re not there? [...] I think there’s always going to be some cases where they’re just not going to manage to do all of it. I mean hopefully maybe in time that that’ll get less and less, but it’s trying to be sensitive to their needs as well, at the same time making sure they realise the importance […] everybody across the patch is working hard and doing their best. (006 Workstream Lead)

## Discussion

It is important to attend to the broader fields of improvement practice (rather than just individuals and groups within local organizations), as these are most often relevant for the scale-up and spread of interventions [4]. The underpinning evidence base for all collaboration-based approaches ‘remains equivocal’ with much still to learn about which components of quality improvement collaboratives are most associated with successful scale up and spread ([9], p14). Existing studies are limited by publication bias, while the functioning of collaboratives as complex interventions remains a ‘black box’ [40, 41]. In this paper, we address concerns about the existing evidence base for PSCs, which are largely underexplored and undertheorized, by explicating the features of the work undertaken by regional leads to support the optimisation care pathway.

A core feature of workstream leads’ work included building and nurturing communities of practice (CoP) across their regions. CoPs are ‘groups of people who share a concern, a set of problems, or a passion about a topic, and who deepen their knowledge and expertise in this area by interacting on an ongoing basis’ [42, p4]. They are increasingly used in healthcare to manage knowledge and improve organisational performance [43]. Workstream leads’ work facilitating CoPs entailed bringing people together across disciplinary boundaries, underpinned by data work [36], to support perinatal optimisation. In line with the diversity in how both PSCs and CoPs are operationalised, activities within the CoPs varied but broadly served the purposes of legitimating the perinatal optimisation pathway and enrolling staff in the delivery of it. Through the CoP, regional leads provided QI expertise, harnessed audit data to prompt action, created safe spaces for problem solving and shared goals, and shared effective implementation strategies across groups. Our findings also draw out the significance of cognitive participation in this context and the relevance of different forms of relational work (initiation, enrolment and legitimation) for building and sustaining CoPs around perinatal optimisation.

Our data also highlight the turbulence and flux involved in regional leads’ daily negotiation and relational practices to support local implementation of the optimisation pathway. May draws attention to implementation processes as non-linear, emergent and dynamic events within systems [35]. However, ‘continuous improvement’ calls to mind a steady upwards trajectory. Our data highlighted the turbulence and flux involved in regional leads’ daily negotiation and relational practices to support local implementation of the optimisation pathway. Turbulencewas shaped by the nature of the pathway, the level of material support provided within the collaboratives, and the wider dynamic context of maternity and neonatal services. As May et al. note ‘how intervention components are coupled to each other and how they are coupled to dynamic elements of context’ has consequences for implementation ([35], p6). At times, regional leads had limited agency or means to adjust fluctuating levels of engagement in collaborative efforts. Ostrom has highlighted the potential for discord between individual and collective interests where there is a ‘commons’ or shared collaborative resource [44]. Previous research has highlighted limits imposed by competing priorities at organisation level [45]; our data showed how national safety priorities added another layer for regional leads to negotiate with. Context is ‘an actor in the drama rather than background’ ([3], p1016). This study was undertaken at a time of intense scrutiny on healthcare safety, particularly in maternity care after a series of high-profile inquiries. The resulting recommendations have arguably led to a shift towards greater public and political demands for accountability and transparency [46, 47]. As Cribb notes, notions of ‘improvement’ in quality and safety are necessarily value laden and link to accountability frameworks; clinical staff are then required to use ‘creative autonomy’ in practice, to decide what constitutes a safety priority [28].

May et al. note that implementation involves negotiations between participants and contexts in relation to ‘the plasticity of intervention components, the degree of participants’ discretion over resource mobilisation and actors’ contributions, and the elasticity of contexts’ [29]. Our analysis highlights the precarity of this type of regional improvement work, particularly when linked to data work [36]. Peaks and troughs in the audit data showing how many eligible women or babies received each element of the pathway were attributed to both shifts in focus and resourcing and to the rhythms of healthcare, such as rotations and new doctor inductions. The number of different elements, spanning antenatal and postpartum care, added to the complexity of the pathway, and affected compliance [48]. This sense of ebb and flow sits at odds with a model of continuous improvement, which guides the approach to pathway implementation. The continuous improvement model can engender a constant sense of chasing unachievable goals, and a focus on elements of practice that are amenable to metrics at the expense of other areas of care [35].

Regional leaders were involved in everyday micro-politics and normative considerations about what constitutes good or ethical practice. We found that this type of work lay outside the NPT coding structure [49], but was important to recognise. Regional leads needed creativity and compassion to work effectively with clinical staff who were often under strain. Regional staff appeared knowledgeable and reflexive, taking account of social structures and what they saw as reasonable and ethical, contributing to the conditions in which communities of practice can emerge and flourish [41]. Our analysis highlighted a layer of hidden emotional work that regional leaders undertook to engage relevant staff with the CoP and maintain their engagement in the context of a healthcare system under pressure. They balanced their own agenda for improving implementation of the pathway with an awareness that stakeholders were juggling multiple priorities and heavy clinical workloads. This showed sensitivity to concerns that QI initiatives might unintentionally negatively impact staff wellbeing, increase burnout and exacerbate workforce shortages [50].

For those commissioning, designing and leading improvement efforts involving PSCs, more attention needs to be paid to the amount of pressure within a system and the need for adaptive change [51]. Sufficient resources need to be allocated to support improvement leads efforts to embed long-term sustainable change [23]. Mandel and Cady highlight how using quality improvement as the main way to change healthcare can limit its impact, particularly if it focuses more on performance targets than on people’s experiences [52]. Our findings draw attention to the valuable work regional leads undertake to take into account staff’s emotional experiences alongside the need to improve optimisation outcomes. This balancing work needs to be valued and taken account of in the design of improvement programmes. Future programmes should utilise models of change that are grounded in organisational and social science, that take account of emotional experience measures, alongside process-technical measures [52].

## Conclusions

Our findings provide an overview of the landscape of perinatal optimisation at regional level in England, helping to open up the ‘black box’ and significance of collaborative efforts managed via national Patient Safety Collaboratives. We demonstrate how efforts for the regional leads in managing external pressures often competed with the goals of the collaborative [9]. Our data highlight the significance of the micropolitical dimensions of regional leads’ improvement work, notably managing precarity and normativity, and raises questions around how best to ensure sustainability of change given the current inelasticity of the implementation environment of maternity and neonatal care. We recommend that policy makers and commissioners value the dual focus on staff emotional wellbeing and the need for improvement in services as an effective way to maximise staff engagement and reach quality improvement goals.

## Supplementary Information

Below is the link to the electronic supplementary material.

Supplementary Material 1

Supplementary Material 2

## Data Availability

The datasets supporting the conclusions of this article are available in the University of Leicester repository https://doi.org/10.25392/leicester.data.c.8336134.
