# Supplementary material for: Explicating the work of regional leads in implementing at scale: a qualitative study of the perinatal optimisation care pathway
Source: Implement Sci Commun. 2026 Aug 6;7:220. doi: 10.1186/s43058-026-01061-3 (PMC13737671; doi:10.1186/s43058-026-01061-3)
Supplement: Supplementary file 1 [file 43058_2026_1061_MOESM1_ESM.docx]

**PREMPATH: Improving the optimisation and stabilisation of the preterm infant: interviews to map the landscape of care provision**

**Topic Guide**

**Scoping interviews: Patient Safety Collaborative Leads**

Look for basic information about the region before the interview and use to personalise the questions.

**Welcome and Introduction**

Seek additional verbal consent to continue and to audio-record the interview

Re-cap of the project and plan for the interview.

Our project aims to evaluate a number of evidence-based interventions intended to improve the optimisation and stabilisation of preterm infants. The scoping interviews will enable us to map and understand the local landscape of care provision in your area. We want to learn how organisations and teams which are involved in the care of preterm infants work together, and how this varies across your region and network. This is not about assessing your region. We just want to understand what is unique about [your region], what works well and what the challenges are.

We don’t expect that you will be able to answer all the questions in depth, and so we will be asking you to signpost us to others who are directly engaged with local services.

# 1: The participant

- How would you like us to refer to you in our outputs? [note in the case of PSC leads, suggest numerical format ‘PSC Lead:01’
- How long have you been in post/ in organisation [x]?
- Can you tell me a little about your background?
  - Clinician/ academic other?
  - Reasons for being involved in the PSCs

# 2: Detail of region

# Can you tell me about the region that your PSC covers?

# Probe for defining features (geographical spread, rural v urban, population, diversity, deprivation)

# Probe for stability and change (e.g. mergers, boundary changes, events)

# 3. Pathway configuration

- Thinking about the 7-element pathway, how is this care pathway of interventions^*^ working in practice in your region? [adapt if more than 7 elements]
  - Probe for elements working well, elements still need work and why
  - Probe for barriers and facilitators
  - Have recent improvement efforts achieved what they intended to do?
- Who is leading the implementation of the 7-element pathway?
  - Probe for whether each element has separate leadership and if/how they are coordinated.
- Does [region] have a parent advisory lead? If so, what is their role?
  - Probe for role of any other parent groups and local charities.

# How is this 7-element pathway working within this region’s PSC and Academic Health Science Network?

# Probe for challenges (e.g. Is this pathway improvement work prioritised? Turnover of staff leading this?)

# What are the QI skills within the PSC, in terms of access to data, types of QI methodology used and how are they used in relation to the implementation of this pathway?)

# 4: Context of service provision

# What are the main opportunities and challenges that services and units are currently dealing with?

# Probe for issues around workload, capacity, cohesion and disparities across region, transfers, change.

# 5: Closing Questions

- Is there anything further that you think we should know about the local pathway?
- Would you be willing to forward details of our project to two or three other key people involved in the local pathway, please?
- Thank you so much for taking the time to speak with me. Before I go, do you have any questions for me?

*place of birth, antenatal steroids, antenatal magnesium sulphate, intrapartum antibiotics, cord management, normothermia and maternal breast milk
