# Supplementary material for: Explicating the work of regional leads in implementing at scale: a qualitative study of the perinatal optimisation care pathway
Source: Implement Sci Commun. 2026 Aug 6;7:220. doi: 10.1186/s43058-026-01061-3 (PMC13737671; doi:10.1186/s43058-026-01061-3)
Supplement: Supplementary file 2 [file 43058_2026_1061_MOESM2_ESM.docx]

SRQR Explicating the work of regional leads in implementing at scale: a qualitative study of enabling perinatal optimisation

| No. | Topic | Page(s) |
| --- | --- | --- |
| **Title and Abstract** | | |
| S1 | Title | 1 |
| S2 | Abstract | 2 |
| **Introduction** | | |
| S3 | Problem formulation | 4-5 |
| S4 | Purpose or research question | 5 |
| **Methods** | | |
| S5 | Qualitative approach and research paradigm | 6 |
| S6 | Researcher characteristics | 6 |
| S7 | Context | 6 |
| S8 | Sampling strategy | 6 |
| S9 | Ethical issues pertaining to human subjects | 6 |
| S10 | Data collection methods | 6 |
| S11 | Data collection instruments and technologies | supplementary |
| S12 | Units of study | 6 |
| S13 | Data processing | 6 |
| S14 | Data analysis | 6 |
| S15 | Techniques to enhance trustworthiness | 6 |
| **Results/findings** | | |
| S16 | Synthesis and interpretation | 7-12 |
| S17 | Links to empirical data | 7-12 |
| **Discussion** | | |
| S18 | Integration with prior work, implications, transferability, and contribution(s) to the field | 12-14 |
| S19 | Limitations |  |
| **Other** | | |
| S20 | Conflicts of interest | 14 |
| S21 | Funding | 14 |
